# Clinical follow-up of left atrial appendage occlusion in patients with atrial fibrillation ineligible of oral anticoagulation treatment—a systematic review and meta-analysis

**DOI:** 10.1007/s10840-021-00953-9

**Published:** 2021-02-13

**Authors:** Frida Labori, Carl Bonander, Josefine Persson, Mikael Svensson

**Affiliations:** Health Economics and Policy, School of Public Health and Community Medicine, Institute of Medicine, University of Gothenburg, Box 463, 405 30 Gothenburg, Sweden

**Keywords:** Atrial fibrillation, Contraindication, Left atrial appendage occlusion, Left atrial appendage closure, Ischemic stroke, Systematic review

## Abstract

**Purpose:**

The recommended stroke prevention for patients with atrial fibrillation (AF) and increased risk of ischemic stroke is oral anticoagulation (OAC). Parts of the patient population are not eligible due to contraindication, and percutaneous left atrial occlusion (LAAO) can then be a preventive treatment option. The aim of this systematic review and meta-analysis is to estimate the long-term clinical effectiveness of LAAO as stroke prevention in patients with AF, increased risk of ischemic stroke, and contraindication to OAC.

**Methods:**

We performed a systematic review and meta-analysis, using Poisson random effect models, to estimate the incidence rate (events per 100 patient-years) of ischemic stroke, transient ischemic attack, major bleeding, and all-cause death after LAAO treatment. We also calculated the risk reduction of ischemic stroke with LAAO compared with no stroke prevention estimated through a predicted risk in an untreated population (5.5 per 100 patient-years).

**Results:**

We included 29 observational studies in our meta-analysis, including 7 951 individuals and 12 211 patient-years. The mean CHA^2^DS^2^-VASc score among the patients in the included studies is 4.32. The pooled incidence rate of ischemic stroke is 1.38 per 100 patient-years (95% CI 1.08; 1.77). According to a meta-regression model, the estimated incidence rate of ischemic stroke at CHA^2^DS^2^-VASc 4 is 1.39 per 100 patient-years. This implies a risk reduction of 74.7% with LAAO compared to predicated risk with no stroke prevention.

**Conclusions:**

Our results suggest that LAAO is effective as stroke prevention for patients with AF, increased risk of stroke, and contraindication to oral anticoagulation.

**Supplementary Information:**

The online version contains supplementary material available at 10.1007/s10840-021-00953-9.

## Introduction

Stroke was globally the second most common cause of death and cause of disability adjusted life years (DALYs) among adults in 2016 [1]. Of all stroke events, approximately 85% are ischemic strokes [2]. A major risk factor for ischemic stroke is atrial fibrillation (AF), which is the most common heart arrhythmia with a prevalence of roughly 3% in the general adult population [3].

Stroke prevention with oral anticoagulation (OAC)^1^ is an important part of the treatment regime in patients with AF. The European Society of Cardiology (ESC) state in their guidelines for AF [3] that treatment with OAC is recommended at CHA^2^DS^2^-VASc scores of ≥2 for men and ≥3 for women. If patients are eligible for non-vitamin K antagonist oral anticoagulant (NOAC), it is preferred over vitamin K antagonist (VKA) [3].

Due to contraindications, parts of the population with AF are not eligible for OAC. According to the ESC guidelines on AF, stroke prevention with percutaneous left atrial appendage occlusion (LAAO)^2^ can be an option for patients with AF and contraindications for OAC (class IIb and level of evidence B), where contraindication is defined as “those with a previous life-threatening bleed without a reversible cause” [3]. LAAO can be conducted with a device that closes the left atrial appendage [4], which is where approx. 90% of thrombi originate in patients with AF [5]. A class IIb recommendation implies that the efficacy is not well-established by evidence and the level of evidence of LAAO is B, which means that data supporting the recommendation is based on data from a single randomized controlled trial (RCT) or non-randomized studies [3].

To our knowledge, there are only two completed RCTs evaluating the efficacy of LAAO: the Watchman Left atrial Appendage Closure Technology for Embolic Protection in Patients With Atrial Fibrillation (PROTECT AF) study [6] and Prospective Randomized Evaluation of the Watchman Left Atrial Appendage Closure device in patients with atrial fibrillation versus long-term warfarin therapy (PREVAIL) [7]. However, in these RCTs, patients were excluded if they had contraindication for long-term OAC, which is problematic when ESC guidelines for AF state that LAAO may be considered for patients with contraindications of OAC [3]. Meanwhile, many (primarily small-scale) non-randomized studies have been conducted that focus on patients with contraindications, which may provide valuable insight in the absence of RCT evidence.

LAAO is associated with a relativity high implementation cost compared to OAC treatment [8]. Resources within the healthcare sector are scarce, and it is crucial to allocate resources in an efficient manner. It is therefore important to estimate the effectiveness of LAAO as stroke prevention in patients with AF and contraindication to OAC. Previously published systematic reviews and meta-analysis [9, 10] of the effects of LAAO do not focus on patients with contraindications, and there is still no consensus on the long-term effectiveness of LAAO as stroke prevention in these patients.

We address this knowledge gap by conducting a systematic review and meta-analysis of the long-term clinical effectiveness of percutaneous endocardial LAAO as stroke prevention in patients with AF, and contraindication to OAC. Specifically, we aim to estimate the incidence rate of ischemic stroke, transient ischemic attack (TIA), major bleeding, and all-cause mortality after the post-procedural period in this population, as well as the risk reduction of ischemic stroke compared to no stroke preventive treatment.

## Method

### Literature search

The main literature search of the systematic review and meta-analysis was conducted in PubMed with complementary searches also conducted using Google Scholar. The applied search term used in PubMed were *atrial appendage AND (occlusion OR closure)* sorted by; *best match*, to generate the greatest number of search results. The search term and process were decided jointly by the lead author of this paper and a medical university librarian. The PubMed search was conducted on October 18, 2019, and the results were screened as follows: as a first step, the lead author screened all titles for potentially relevant studies, followed by reviewing the abstracts belonging to these titles. As a last step, articles were reviewed in full-text, and the final decision of eligibility for inclusion in the systematic review and meta-analysis was made on the full-text article. The search terms used in Google Scholar were *left atrial appendage occlusion* and *left atrial appendage closure*. Each term was searched separately, and limited to studies published 2010 or later. This limitation was made since in the initial PubMed search, no studies published before 2012 were included in the systematic review and meta-analysis, and to decrease the number of search results in the Google scholar searches. We screened the 200 first search results in each search, and if any new titles were relevant abstracts were read continuously. We also screened the reference list of the studies included in the systematic review and meta-analysis for additional studies.

Inclusion criteria were long-term follow-up of percutaneous endocardial LAAO and inclusion of patients with contraindication for OAC. Reasons for exclusion were, for example, meta-analysis, systematic reviews, epicardial interventions, specific subpopulations such as patients with kidney disease or heart failure, and follow-up less than 11 months, since our aim is to study long-term effects from approximately 1 year and onwards. Furthermore, studies were excluded if we suspected overlapping study populations, and the articles most recently published or largest sample size were included. A full list of exclusion criteria is available in the Supplementary information 1.

### Data extractions

One person extracted data from the original articles manually. Data were extracted into four main categories: study characteristics, patient characteristics, device, and outcomes. Each main category includes several variables, presented in the Supplementary information 2.

Patient characteristics such as age and CHA^2^DS^2^-VASc score were extracted from the original articles as mean and standard deviations (SD) when reported, and median and interquartile range (IQR) as a secondary choice. For the variables gender, procedural success, ischemic stroke, TIA, major bleeding, and all-cause death, data were extracted in absolute numbers. Data on outcome variables were included if events occurred >7 days after procedure, i.e., excluding peri-procedural and short-term adverse events. Furthermore, when possible data were only included from patients having a successful procedure, e.g., patients received a LAAO device. However, we do not believe that this would have any large impact on the results in our analysis, since the average procedural success rate in our systematic review and meta-analysis was 97.3%.

The length of follow-up was extracted as mean follow-up and SD if reported in the original articles and median and IQR as a second choice. A few studies did not present either mean or median follow-up in their articles. In these cases, we assumed that the mean follow-up was 12 months, since these studies were all presented as 1-year follow-up studies. To be able to estimate the incidence rate of health outcomes after LAAO, the total number of patient-years were extracted. The number of patient-years was applied from the original article if it was reported. For the articles that did not report the number of patient-years, we estimated the number of patient-years by multiplying the number of patients with successful procedure with mean follow-up time.

### Quality assessment

Quality assessment of the individual studies was carried out with an adjusted version of the Newcastle-Ottawa scale (NOS) for cohort studies [11]; detailed information is available in the Supplementary information 3.

### Statistical analysis

Descriptive statistics for the original articles included in the systematic review and meta-analysis is presented as means weighted by sample size.

We conducted a meta-analysis to estimate pooled incidence rates and 95% confidence interval (CI) for each outcome. Our outcome measures reflect numbers of events (counts) per patient-year. In our main analysis, we used random effects Poisson regression to estimate pooled incidence rates to account for potential heterogeneity between studies. The dependent variable was the number of events (ischemic stroke, TIA, major bleeding, and all-cause death), while the logarithm of the total number of patient-years was used as explanatory variable. Between-study heterogeneity was measured using *I*^2^ [12].

Friberg et al. [13] present predicted risk of ischemic stroke per 100 patient-years at different CHA^2^DS^2^-VASc scores, based on a cohort of 90,490 patients with AF without VKA treatment in Sweden. The predicted risk for ischemic stroke for a patient with CHA^2^DS^2^-VASc score of 4 and no stroke prevention is 5.5 per 100 patient-years [13].

We calculated the risk reduction with LAAO as stroke prevention compared to the predicted risk of ischemic stroke with no stroke prevention by Friberg et al. [13]. The mean CHA^2^DS^2^-VASc score in our meta-analysis sample is 4.32. To be able to compare the pooled incidence rate of ischemic stroke from our meta-analysis among patients treated with LAAO with the predicted risk score for patients without stroke prevention (5.5 per 100 patient-years), we adjusted our pooled estimate to reflect patients with CHA^2^DS^2^-VASc score 4. This was done using Poisson meta-regression with CHA^2^DS^2^-VASc score as a covariate.

Statistical analyses were carried out using the statistical analysis packages Stata (version 16) and R (version 3.6.3).

### Sensitivity analysis

As a sensitivity analysis, we estimated the pooled incidence rate using the inverse variance method for random effects. In this analysis, we used a continuity correction that added 0.5 events to studies that had no events. To identify and asses potential publication bias, we constructed funnel plots for each outcome. In case of asymmetry in the funnel plots, we used the trim and fill method to minimize that influence of extreme values (trim) and fill in with hypothetical values to create symmetry in the funnel plot [12, 14].

### Heterogeneity

To identify potential causes of heterogeneity between studies, we conducted a Poisson meta-regression for each outcome measure with study-level characteristics as covariates.

### Ethical considerations

No ethical approval was sought for this systematic review and meta-analysis, since this study design only includes secondary data, and there is none/minimal risk of causing any harm to the patients in the original studies.

## Results

### Literature search and data collection

**Fig. 1:**
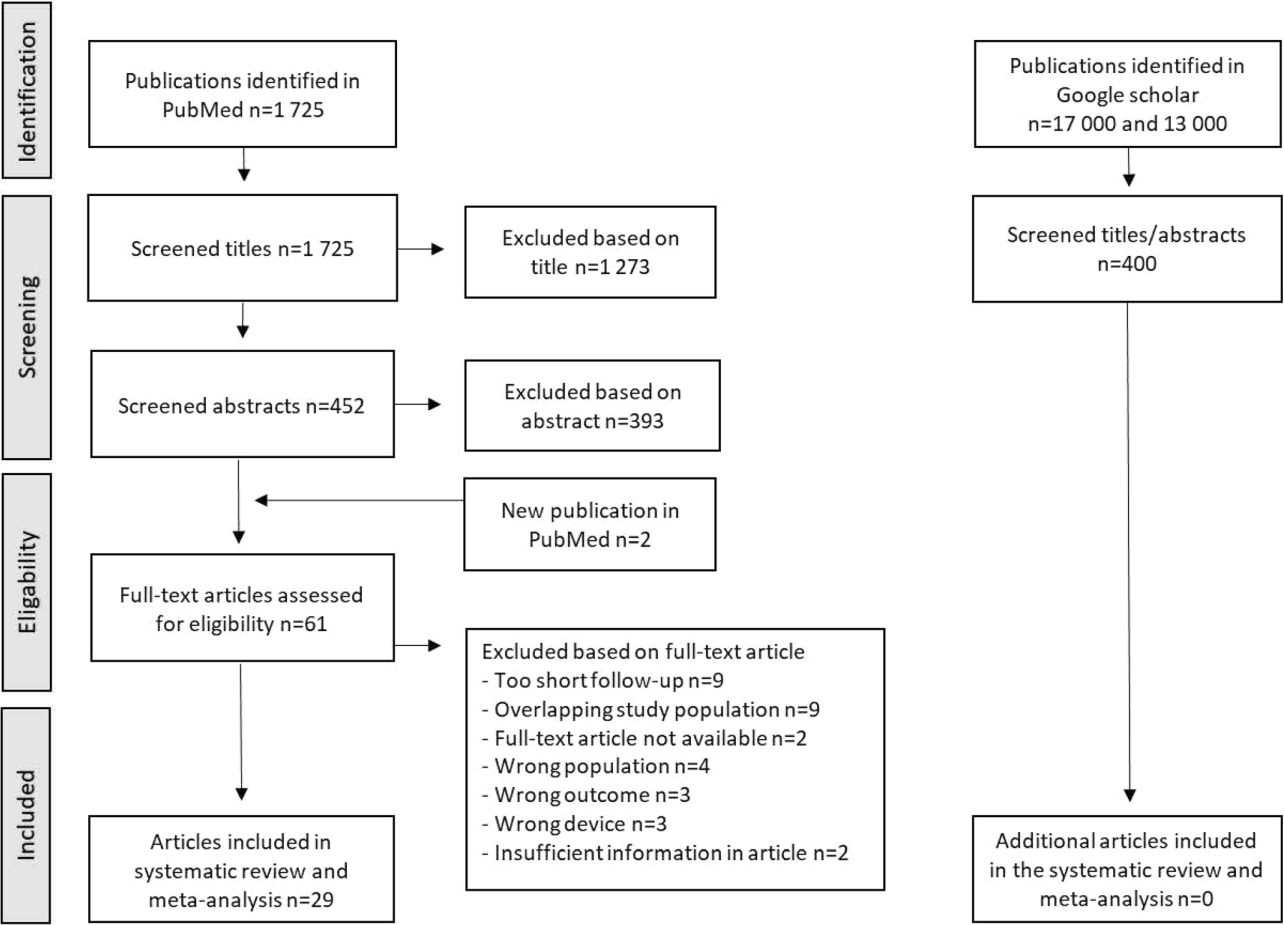
PRISMA flowchart of the reviewing process. PRISMA, Preferred Reporting Items for Systematic Review and Meta-analyses

Twenty-nine articles were included in the systematic review and meta-analysis. The process of identification, screening, and inclusion is illustrated in a PRISMA flowchart (Fig. 1).

### Study population

Pooling the data from the 29 included articles [15–42] resulted in a study population of 7951 individuals and with a weighted mean of 1.46 years of follow-up and a total of 12,211 patient-years. The average CHA^2^DS^2^-VASc and HAS-BLED scores were 4.32 and 3.19 respectively, which both indicate an increased risk for ischemic stroke and bleedings. In the study population included in the meta-analysis, 37.5% previously had a stroke and 60.3% had a major bleeding on average. The mean age in the included articles ranges from 64.4 years [25] to 79.6 [28] years in the meta-analysis. Detailed information on the included articles is available in Supplementary information 4.

LAAO devices used in the included articles include the Amplatzer cardiac plug (ACP), Amplatzer amulet (Abbott medical), Watchman (Boston scientific), WaveCrest (Coherex medical), and LAmbre (Lifetech scientific).

The post-procedural treatment after LAAO differs between and within the included studies. In the majority of the studies, patients were treated with a dual antiplatelet treatment (DAPT) for 1–6 months, followed by life-long single antiplatelet treatment (APT).

### Ischemic stroke and TIA

**Fig. 2:**
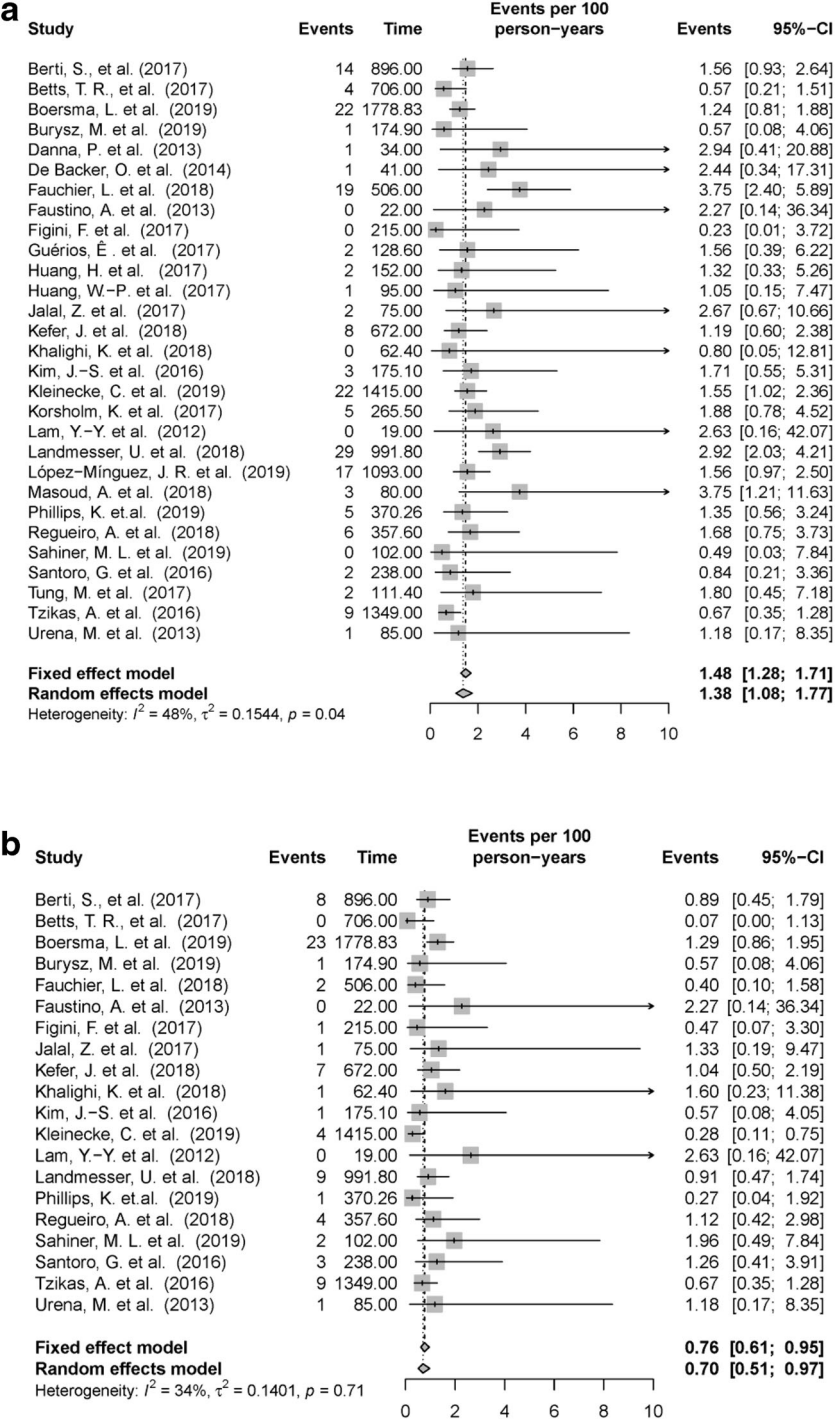
Forest plot illustrating incidence rate (95% CI) for ischemic stroke (**a**) and TIA (**b**) in the individual studies and the pooled incidence rate from the Poisson random effect model. For individual studies with zero event, 0.5 continuity corrections are applied to calculate the individual incidence rate. *CI*, confidence interval

The number of ischemic strokes was reported in all included articles, and the number of TIAs was reported in 20 out of 29 articles (Fig. 2). The lowest incidence of ischemic stroke observed in the included articles was 0 events [22, 28, 32, 38, 43] and the highest incidence rates observed were 3.8 per 100 patient-years [21] (Fig. 2). The random effects Poisson model resulted in a pooled incidence rate of 1.38 and 0.70 per 100 patient-years for ischemic stroke and TIA, respectively (Table 1). The incidence rates of ischemic stroke and TIA are not associated with any considerable heterogeneity (*I*^2^ = 48% and 34%).

**Table 1:** Random effects incidence rate, from Poisson and inverse variance method

Variable	*N* observations	Poisson analysis incidence rate* (95% CI)	Heterogeneity (*I*^2^)	Inverse variance analysis incidence rate* (95% CI)	Heterogeneity (*I*^2^)
Ischemic stroke	29	1.38 (1.08; 1.77)	48%	1.58 (1.27; 1.97)	39%
TIA	20	0.70 (0.51; 0.97)	34%	0.91 (0.73; 1.15)	2.5%
Major bleeding	27	2.22 (1.58; 3.13)	86%	2.64 (1.97; 3.55)	81%
All-cause mortality	27	4.38 (3.26; 5.89)	91%	5.46 (4.47; 6.66)	80%

*Incidence rate per 100 patient-years

**Fig. 3:**
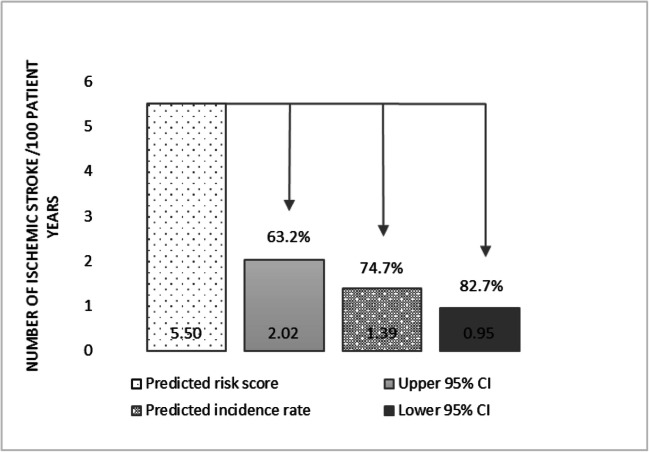
Risk reduction of ischemic stroke in patients with CHA^2^DS^2^-VASc 4, predicted risk score versus predicted incidence rate and lower and upper bound of the confidence interval and the predicted incidence rate.

The predicted incidence rate of ischemic stroke after LAAO for patients with a CHA^2^DS^2^-VASc score of 4, estimated in the meta-regression, is 1.39 (95% CI: 0.95; 2.02). When comparing this predicted incidence rate to the predicted risk score at CHA^2^DS^2^-VASc 4 (5.5 per 100 patient-years), this implies a 74.7% decrease in the risk of ischemic stroke with LAAO compared to no stroke prevention. When we calculate the risk reduction between the predicted risk of ischemic stroke [13] and the lower and upper bound of the confidence interval, LAAO decreases the risk of ischemic stroke by 82.7% and 63.2% respectively (Fig. 3).

### Major bleeding

**Fig. 4:**
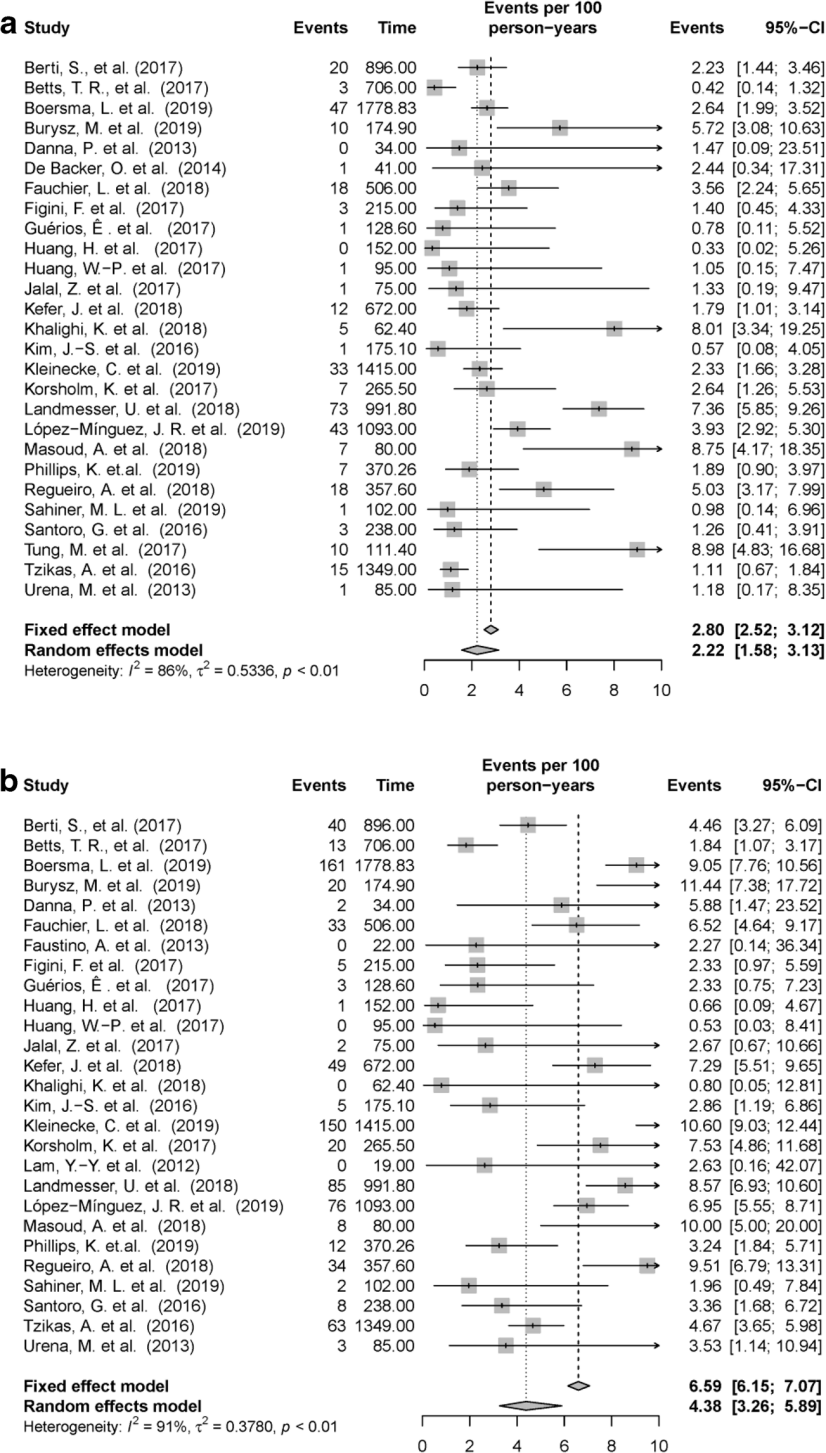
Forest plot illustrating incidence rate (95% CI) for major bleeding (**a**) and all-cause mortality (**b**) in the individual studies and the pooled incidence rate from the Poisson random effect model. For individual studies with zero event, 0.5 continuity corrections are applied to calculate the individual incidence rate. *CI*, confidence interval

Major bleeding was reported in 27 of the 29 included articles, with incidence rates ranging from zero major bleedings [19, 24] to 8.98 major bleedings per 100 patient-years (Fig. 4) [40]. The pooled incidence rates from random effects Poisson model were 2.22 events per 100 patient-years (Table 1). There was large between-study heterogeneity in the incidence of major bleeding (*I*^2^ = 86%).

### All-cause death

Out of the 29 included studies, 27 reported the number of deaths in their studies. The incidence rates of all-cause death ranged from zero [22, 25, 28, 32] to 11.4 events per 100 patient-years [18] (Fig. 4). In the random effects Poisson model, the pooled incidence rates of all-cause death were 4.38 events per 100 patient-years (Table 1). There was large between-study heterogeneity in all-cause mortality (*I*^2^ = 91%).

### Sensitivity analysis

We used an inverse variance method as a sensitivity analysis; for the outcome measures ischemic stroke, TIA, and major bleeding, there were no considerable differences from the main analysis. Detailed information from the inverse variance analysis is presented in Table 1.

**Fig. 5:**
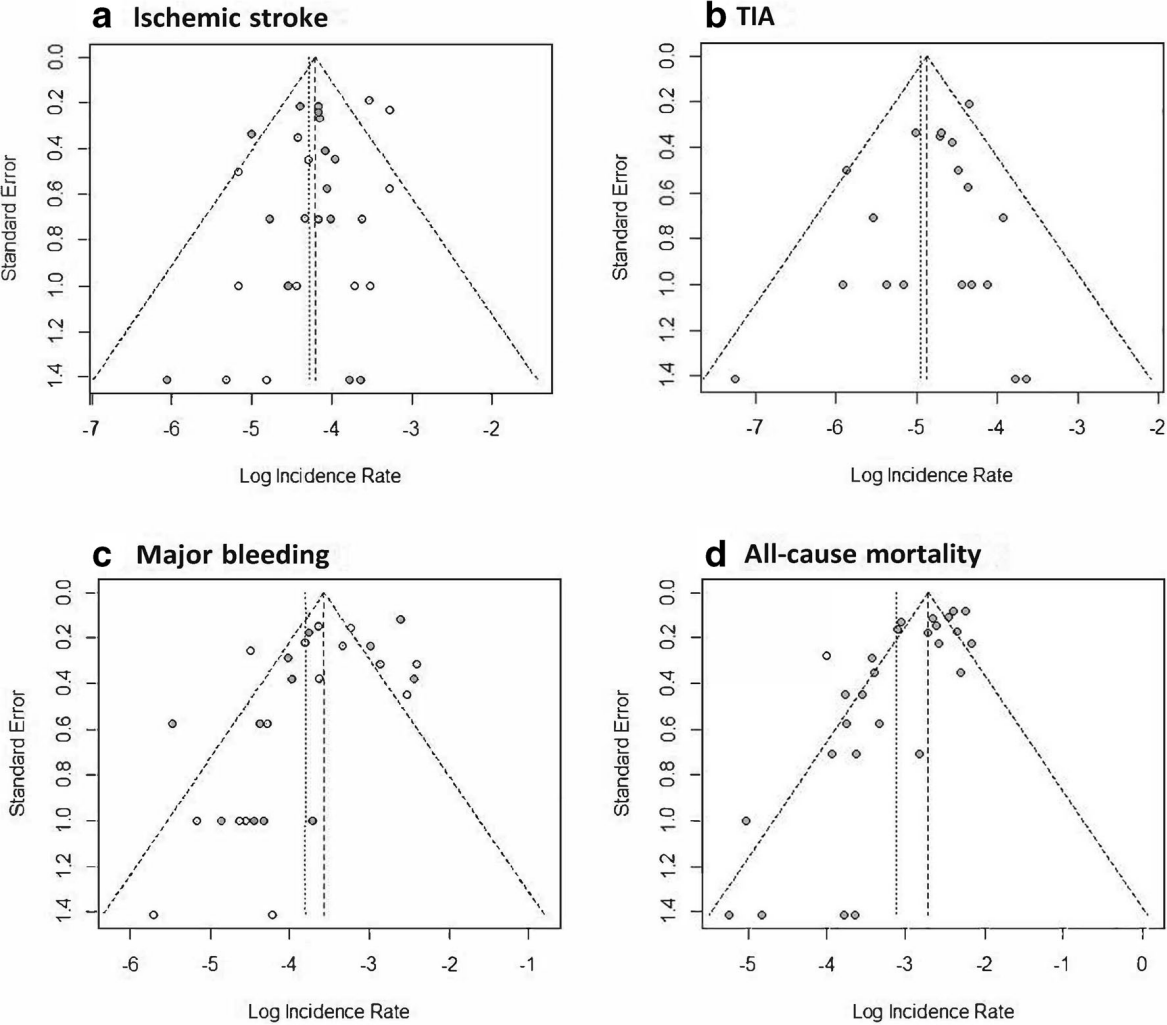
Funnel plots illustrates potential publication bias

Ischemic stroke, major bleeding, and all-cause mortality have noticeable asymmetry in their funnel plots (Fig. 5). Furthermore, both major bleeding and all-cause mortality have no studies in the lower right quadrant of the figure, i.e., studies with higher-than-average incidence of the event and large standard errors, which can indicate that there is a case of an existing publication bias. We therefore conducted a trim-and-fill analysis by adding potentially missing studies to reach symmetry of the funnel plots for ischemic stroke, major bleeding, and all-cause death. The result from the trim-and-fill sensitivity analysis do not differ substantially from the main results. Detailed information from trim-and-fill analysis is available in the Supplementary information 5.

### Heterogeneity

We conducted a meta-regression to identify potential causes of between-study heterogeneity in incidence rates of major bleeding and all-cause death. Covariates that significantly influenced the between-study heterogeneity in studies reporting major bleeding were mean age and proportion of patients with previous major bleeding, which were associated with increased incidence rates. For all-cause mortality, the sample size and publication year were positively correlated with mortality rates. Detailed results from the meta-regression is available in the Supplementary information 6.

## Discussion

We have conducted a systematic review and meta-analysis of the long-term clinical effectiveness of LAAO as stroke prevention for patients with AF, and contraindication for OAC. Our result indicates that LAAO is effective in preventing ischemic stroke in patients with AF that have a contraindication to OAC, with a 74.7% risk reduction of ischemic stroke after LAAO compared to the predicted ischemic stroke rate in a no stroke prevention population at CHA^2^DS^2^-VASc 4. Furthermore, we estimated the incidence rates of major bleeding and all-cause mortality after LAAO, which were 2.22 per 100 patient-years and 4.38 per 100 patient-years each.

The estimated effect size is difficult to compare directly to those from existing clinical trials (PROTECT AF/PREVAIL), as they only included patients without contraindications and compared LAAO to treatment with VKA (instead of no stroke prevention). We can, however, compare the incidence rates in our meta-analysis to the treatment arms in the PROTECT AF/PREVAIL trials that received a LAAO device, to assess if our pooled incidence rates are comparable to the only existing clinical trials. The pooled incidence rate in our meta-analysis is 1.38 ischemic strokes per 100 patient-years after LAAO. This result is consistent with the combined 5-year outcomes of the PROTECT AF and PREVAIL trials [44], where the incidence rate of ischemic stroke and systemic embolism was 1.3 per 100 patient-years after LAAO followed by 45 days of VKA and 6 months of dual antiplatelet treatment (DAPT) in patients without contraindications [44].

Furthermore, our result is also comparable to previous published systematic reviews and meta-analysis [9, 10], even though these are not focusing on patients contraindicated to OAC. In a recently published systematic review and meta-analysis focusing on the expected versus the observed rate of ischemic stroke after LAAO [45], it reported a pooled risk reduction of ischemic stroke of 73.6% (95% CI, 68.9 to 78.3%) [45]. Their result is in line with 74.7% risk reduction estimated in our study for patients with CHA^2^DS^2^-VASc 4, compared to the predicted risk according to Friberg et al. [13]. To our knowledge, our systematic review and meta-analysis is the first focusing on contraindicated patients only, which is the patients that should be considered for LAAO according to the ESC guidelines [3].

There is an ongoing RCT (clinicaltrials.gov, NCT02928497) that is investigating the effectiveness of LAAO in patients with contraindication, but no results are available yet. In the meantime, our systematic review and meta-analysis contribute with valuable knowledge on the clinical effectiveness of LAAO in patients with contraindications. Thus, the findings in our study can guide policy-makers to make evidence-based decision-making when evaluating and planning treatment strategies for patients with AF, increased risk of ischemic stroke, and contraindication for OAC.

### Limitations

Due to a lack of controlled trials, the studies included in our systematic review and meta-analysis are observational studies without any control group, and we therefore compare our estimated incidence rate with a predicted risk score for ischemic stroke [12]. The quality of the available evidence could be strengthened by RCTs that focus on patients with contraindication to OAC and compare LAAO with other stroke preventive interventions.

In regard to the risk reduction of ischemic stroke with LAAO compared to no stroke prevention, the latter is based on a predicted risk score, based on a cohort of 90,490 patients with AF in Sweden. According to Friberg et al. [13], the predicted risk of ischemic stroke at CHA^2^DS^2^-VASc 4 is 5.5 per 100 patient-years without stroke prevention. In their article [13], there is no information about the uncertainty of the predicted value. Thus, the uncertainty estimates for the effectiveness of LAAO presented in this paper are based only on the statistical uncertainty associated with the predicted incidence rate for ischemic stroke at CHA^2^DS^2^-VASc 4.

It is important to consider the generalizability of the results presented. Our analysis included studies from different continents that report long-term outcomes after LAAO for patients with AF, increased risk of ischemic stroke, and contraindication to OAC. This is the patient population that, according to the ESC guidelines is the patient population, is to be considered for LAAO treatment [3]. We note that the average CHA^2^DS^2^-VASc score in the included studies was 4.32 (range: 3.6 to 5.0), which implies a high-risk study population. It is unclear if the results are generalizable to populations with lower risk. This is important to keep in mind if the composition of the patient population changes in the future or if other patient populations are recommended LAAO treatment.

Our overall aim was to investigate the long-term clinical effectiveness of LAAO as stroke prevention in patients with AF and contraindication to OAC. While beyond the scope of our study, there are several clinically relevant questions that warrant additional attention in future research. For instance, it is important to investigate how the clinical effectiveness differs between different percutaneous endocardial LAAO devices, and to study which post-procedural treatments that are most effective at reducing the risk of device-related thrombosis. To our knowledge there is currently no consensus regarding which post-procedural treatments that are most efficient after LAAO. Another clinically important question is if there are differences in the timing of when patients are at greatest risk of having, for example, an ischemic stroke after LAAO treatment and how it can be prevented.

## Conclusion

Left atrial appendage occlusion is effective in preventing ischemic stroke in patients with AF, increased risk of ischemic stroke, and contraindication to OAC. We estimate that the risk of ischemic stroke for a patient with CHA^2^DS^2^-VASc 4 is 74.7% lower compared with no stroke prevention. Our results imply that LAAO is equally effective as stroke prevention in patients with contraindications as those patients without contraindications; these findings contribute with valuable input for policy-makers deciding on treatment strategies.

## Supplementary Information

ESM 1 (DOCX 12 kb)

ESM 2 (DOCX 12 kb)

ESM 3 (DOCX 15 kb)

ESM 4 (DOCX 16 kb)

ESM 5 (DOCX 12 kb)

ESM 6 (DOCX 16 kb)

## Data Availability

Available upon request

## Footnotes

1 Oral anticoagulation (OAC) is used as an umbrella expression for both non-vitamin K antagonist oral anticoagulant (NOAC) and vitamin K antagonist (VKA )

2 LAAO in this study refers to percutaneous occlusion of left atrial appendage through different LAAO devices. In the scientific literature, LAAO can also be referred to as left atrial appendage closure (LAAC).

## Code availability

Available upon request
