# Supplementary material for: Clinical follow-up of left atrial appendage occlusion in patients with atrial fibrillation ineligible of oral anticoagulation treatment—a systematic review and meta-analysis
Source: J Interv Card Electrophysiol. 2021 Feb 13;61(2):215–25. doi: 10.1007/s10840-021-00953-9 (PMC8324592; doi:10.1007/s10840-021-00953-9)
Supplement: Supplementary file 1 — (DOCX 12 kb) [file 10840_2021_953_MOESM1_ESM.docx]

**Online Resource 1.** Exclusion criteria’s

| Exclusion criteria |
| --- |
| PROTECT-AF studies |
| PREVAIL studies |
| PLAATO studies |
| Systematic reviews |
| Meta-analysis |
| Intraoperative studies |
| Technical articles (describing procedure or comparing CT. ECG methods) |
| Case-reports |
| Follow-up less than 11 months |
| Surgical LAAO |
| Commentary |
| Posters or conference abstracts |
| Combined interventions |
| Epicardial interventions (devices or sutures) |
| Consensus documents. guidelines |
| Canine models |
| Thoracoscopic LAAO |
| Combined heart surgery (ablation. aorta valve. TAVI. coronary bypass) |
| Thrombus in LAA |
| Cost-effectiveness studies |
| Specific patient populations i.e. only patients with renal disease or chronic heart failure |
| Patient populations stratified by baseline characteristics |
| Operators learning curve |
| Post-operative treatment after LAAO |
| Studies on device leaks |
| Studies on device related thrombus |
| Location of the device disc |
| Subpopulations from other studies |
| Studies were there is newer publications (longer follow-up) |
| Full-text article not available in English, Swedish or Norwegian. |
| Study protocols |
| Overlapping study populations |
| Full-text not available |
| Insufficient information in the article |
