# Supplementary material for: Clinical follow-up of left atrial appendage occlusion in patients with atrial fibrillation ineligible of oral anticoagulation treatment—a systematic review and meta-analysis
Source: J Interv Card Electrophysiol. 2021 Feb 13;61(2):215–25. doi: 10.1007/s10840-021-00953-9 (PMC8324592; doi:10.1007/s10840-021-00953-9)
Supplement: Supplementary file 2 — (DOCX 12 kb) [file 10840_2021_953_MOESM2_ESM.docx]

**Online Resource 2.** Categories and variables extracted from original articles

| Study characteristics | Patient characteristics | Device | | Outcomes |
| --- | --- | --- | --- | --- |
| Author | Age | Device used | | Procedural success |
| Publishing year | Gender |  | Post-procedural treatment | |
| Study design | CHA^2^DS^2^-VASc score |  | Ischemic stroke | |
| Inclusion criteria | HAS-BLED |  | TIA | |
| Aim | Previous stroke |  | Major bleeding | |
| Study population | Previous bleeding |  | All-cause death | |
| Patient-years | Indication for LAAO |  |  | |
| Length of follow-up |  |  |  | |
