# Supplementary material for: Clinical follow-up of left atrial appendage occlusion in patients with atrial fibrillation ineligible of oral anticoagulation treatment—a systematic review and meta-analysis
Source: J Interv Card Electrophysiol. 2021 Feb 13;61(2):215–25. doi: 10.1007/s10840-021-00953-9 (PMC8324592; doi:10.1007/s10840-021-00953-9)
Supplement: Supplementary file 3 — (DOCX 15 kb) [file 10840_2021_953_MOESM3_ESM.docx]

**Online Resource 3.** Quality assessment of the individual studies included in the systematic review and meta-analysis according to the NOS.

|  |  | Selection | |  | Outcome | | |  |  |
| --- | --- | --- | --- | --- | --- | --- | --- | --- | --- |
| Author (year) |  | Representativeness of the exposed cohort | Ascertainment of exposure |  | Assessment of outcome | Was follow-up long enough for outcome to occur | Adequacy of follow up of cohorts |  | Total score |
| Berti (2017) |  | * | * |  | * | * | * |  | 5/5 |
| Betts (2017) |  | * | * |  | * | * |  |  | 4/5 |
| Boersma (2019) |  | * | * |  | * | * |  |  | 4/5 |
| Burysz (2019) |  | * | * |  | * | * | * |  | 5/5 |
| Danna (2013) |  | * | * |  | * | * |  |  | 4/5 |
| De Backer (2014) |  | * | * |  | * | * |  |  | 4/5 |
| Fauchier (2018) |  | * | * |  | * | * |  |  | 4/5 |
| Faustino (2013) |  | * | * |  | * | * |  |  | 4/5 |
| Figini (2017) |  | * | * |  | * | * | * |  | 5/5 |
| Guérios (2017) |  | * | * |  | * | * | * |  | 5/5 |
| Huang^a^ (2017) |  | * | * |  | * |  |  |  | 3/5 |
| Huang^b^ (2017) |  | * | * |  | * | * | * |  | 5/5 |
| Jalal (2017) |  | * | * |  | * | * | * |  | 5/5 |
| Kefer (2018) |  | * | * |  | * | * | * |  | 5/5 |
| Khalighi (2018) |  | * | * |  | * | * |  |  | 4/5 |
| Kim (2016) |  | * | * |  | * | * |  |  | 4/5 |
| Kleinecke (2019) |  | * | * |  | * | * |  |  | 4/5 |
| Korsholm (2017) |  | * | * |  | * | * |  |  | 4/5 |
| Lam (2012) |  | * | * |  | * | * |  |  | 4/5 |
| Landmesser (2018) |  | * | * |  | * |  | * |  | 4/5 |
| López-Mínguez (2019) |  | * | * |  | * | * |  |  | 4/5 |
| Masoud (2018) |  | * | * |  | * | * |  |  | 4/5 |
| Phillips (2019) |  | * | * |  | * | * |  |  | 4/5 |
| Regueiro (2018) |  | * | * |  | * | * |  |  | 4/5 |
| Şahiner (2019) |  | * | * |  | * | * |  |  | 4/5 |
| Santoro (2016) |  | * | * |  | * | * |  |  | 4/5 |
| Tung (2017) |  | * | * |  | * | * |  |  | 4/5 |
| Tzikas (2016) |  | * | * |  | * | * | * |  | 5/5 |
| Urena (2013) |  | * | * |  | * | * |  |  | 4/5 |
