# Supplementary material for: Clinical follow-up of left atrial appendage occlusion in patients with atrial fibrillation ineligible of oral anticoagulation treatment—a systematic review and meta-analysis
Source: J Interv Card Electrophysiol. 2021 Feb 13;61(2):215–25. doi: 10.1007/s10840-021-00953-9 (PMC8324592; doi:10.1007/s10840-021-00953-9)
Supplement: Supplementary file 4 — (DOCX 16 kb) [file 10840_2021_953_MOESM4_ESM.docx]

**Online Resource 4.** Baseline characteristics of the individual studies included in the systematic review and meta-analysis.

| Author | Year | Country | Patient-years | Mean age±SD | CHA^2^DS^2^-VASc | HAS-BLED | History of stroke | History of bleeding |
| --- | --- | --- | --- | --- | --- | --- | --- | --- |
| Berti, S., et al. | 2017 | Italy | 896 | 75.1±8 | 4.2±1.5 | 3.2±1.1 | 27.7% | 41.6% |
| Betts, T. R., et al. | 2017 | United Kingdom | 706 | 72.9±8.3 | 4.2±1.6 | 3.3±1.2 | 49.6% | 70.2% |
| Boersma, L. et al. | 2019 | Multinational | 1778.8 | 73.4±8.9 | 4.5±1.6 | 2.3±1.2 | 30.5% | 31.3% |
| Burysz, M. et al. | 2019 | Poland | 174.9 | 72±8.4 | 5.0±1.5 | 4.4±0.9 | 39.2% | 91.7% |
| Danna, P. et al. | 2013 | Italy | 34* | 73.4±8.3 | 4.3±1.45 | 3.56±1.12 | 16.2% | 45.9% |
| De Backer, O. et al. | 2014 | Denmark | 41* | 74.6±8.2 | 4.5±1.4 | 3.7±0.9 | NR | NR |
| Fauchier, L. et al. | 2018 | France | 507* | 74.9±8.9 | 4.5±1.4 | 3.7±1.0 | 41.1% | 90.1% |
| Faustino, A. et al. | 2013 | Portugal | 22* | 70±9 | 4.7±1.4 | 3.7±1.3 | NR | NR |
| Figini, F. et al. | 2017 | Italy | 215* | 72±9 | 3.9±1.7 | 3.6±1.4 | 29.1% | 60% |
| Guérios, Ê. et al. | 2017 | Brazil | 128.6 | 73.1±10.1 | 4.5±1.5 | 3.6±1.0 | 49.5% | 61.5% |
| Huang, H. et al. | 2017 | China | 152* | 69.3±9.4 | 4.0±1.7 | NR | 64.7% | NR |
| Huang, W.-P. et al. | 2017 | China | 95* | 64.2±8.6 | 3.6±1.6 | NR | 38.7% | NR |
| Jalal, Z. et al. | 2017 | France | 75 | 73±8 | 4.4±1.3 | 3.4±0.9 | 52% | 86% |
| Kefer, J. et al. | 2018 | Belgium | 672 | 75±12 | 4±0 | 3.5±0.7 | 44% | 80% |
| Khalighi, K. et al. | 2018 | USA | 62.4* | 79.6±6.17 | 4.9±1.3 | 3.7±0.9 | 28.6% | 58.3% |
| Kim, J.-S. et al. | 2016 | Korea | 175.1 | 65.1±9.4 | 3.9±1.6 | 2.7±1.3 | 43.8% | NR |
| Kleinecke, C. et al. | 2019 | Germany & Switzerland | 1415 | ACP 74.4±9.9 Amulet:74.4±9.1 | ACP:4.4±1.6 Amulet:4.6±1.7 | ACP:3.2±1.1 Amulet:3.2±0.9 | 32.6% | NR |
| Korsholm, K. et al. | 2017 | Denmark | 265.5 | 73.2±9.9 | 4.4±1.6 | 4.1±1.1 | 50.5% | 82.2% |
| Lam, Y.-Y. et al. | 2012 | China & Australia | 19* | 68±9 | NR | NR | 30% | 50% |
| Landmesser, U. et al. | 2018 | Multinational | 991.8* | 75±9 | 4.2±1.6 | 3.3±1.1 | 39% | 72% |
| López-Mínguez, J. R. et al. | 2018 | Spain & Portugal | 1093 | 75.4** | 4.4±1.5 | 3.4±1.2 | 31.4% | 73.7% |
| Masoud, A. et al. | 2018 | United Kingdom | 80 | 76±8.2 | 4±1 | 3±1 | 77.1% | 95.2% |
| Phillips, K. et.al. | 2019 | Multinational | 370.26* | 70.8±9 | 3.9±1.7 | 2.1±1.2. | 30.8% | NR |
| Regueiro, A. et al. | 2018 | Spain | 357.6 | 75** | 4.8±1.6 | 4.2±0.9 | 41.6% | 82.2% |
| Şahiner, M. L. et al. | 2019 | Turkey | 102* | 72.3±20.1 | 4.6±2.6 | 4.3±3.3 | 28.3% | 88.3% |
| Santoro, G. et al. | 2016 | Italy | 238 | 76.6±7.6 | 4(2-5)** | 3(2-3)** | NR | 40% |
| Tung, M. et al. | 2017 | Australia | 111.4 | 74.9±8.9 | 4.5±1.4 | 3.0±0.95 | NR | 81% |
| Tzikas, A. et al. | 2015 | Multinational | 1349 | 75±8 | 4.5±1.6 | 3.1±1.2 | 39% | 47% |
| Urena, M. et al. | 2013 | Canada | 85* | 74±8 | 5(4-6)** | 4(3-4)** | 61.5% | 90.4% |

*Number of patient-years are not presented in the original article, and therefore the number of patient-years is estimated through mean follow-up time and study participants.
**Median (IQR)
NR= Not reported in original article
