# Supplementary material for: Clinical follow-up of left atrial appendage occlusion in patients with atrial fibrillation ineligible of oral anticoagulation treatment—a systematic review and meta-analysis
Source: J Interv Card Electrophysiol. 2021 Feb 13;61(2):215–25. doi: 10.1007/s10840-021-00953-9 (PMC8324592; doi:10.1007/s10840-021-00953-9)
Supplement: Supplementary file 5 — (DOCX 12 kb) [file 10840_2021_953_MOESM5_ESM.docx]

**Online Resource 5.** Results from the trim-and-fill analysis.

| Variable | Total number of observations | | Added studies | Random effect  incidence rate* (95% CI) | Heterogeneity (I^2^) |
| --- | --- | --- | --- | --- | --- |
| Ischemic stroke | 36 | 7 | | 1.86 (1.46; 2.36) | 49.8% |
| TIA | 21 | 1 | | 0.92 (0.72; 1.17) | 12.4% |
| Major bleeding | 37 | 10 | | 3.74 (2.65; 5.29) | 82.1% |
| All-cause mortality | 38 | 11 | | 7.13 (5.31; 9.58) | 81.4% |

*Incidence rate per 100 patient-years
