# Supplementary material for: Clinical follow-up of left atrial appendage occlusion in patients with atrial fibrillation ineligible of oral anticoagulation treatment—a systematic review and meta-analysis
Source: J Interv Card Electrophysiol. 2021 Feb 13;61(2):215–25. doi: 10.1007/s10840-021-00953-9 (PMC8324592; doi:10.1007/s10840-021-00953-9)
Supplement: Supplementary file 6 — (DOCX 16 kb) [file 10840_2021_953_MOESM6_ESM.docx]

**Online Resource 6.** Addressing causes of heterogeneity using Poisson meta-regression

| Variable | Unadjusted IRR | 95% confidence interval | P-value | Adjusted IRR | 95% confidence interval | P-value |
| --- | --- | --- | --- | --- | --- | --- |
| TIA |  |  |  |  |  |  |
| Years of follow-up | 1.088 | 0.745; 1.591 | 0.662 | 1.167 | 0.675;2.016 | 0.581 |
| Age | 1.114 | 0.939; 1.321 | 0.215 | 1.119 | 0.858;1.458 | 0.406 |
| History of ischemic stroke | 0.113 | 0.002; 8.175 | 0.318 | 0.037 | 0.000; 16.774 | 0.291 |
| History of bleeding | 0.736 | 0.199; 2.717 | 0.645 | 1.643 | 0.152; 17.771 | 0.683 |
| Sample size | 1.000 | 0.999; 1.001 | 0.489 | 1.001 | 0.999; 1.002 | 0.398 |
| CHA^2^DS^2^-VASc | 1.623 | 0.636; 4.143 | 0.311 | 1.333 | 0.423; 4.204 | 0.624 |
| Publication year | 1.027 | 0.834; 1.313 | 0.834 | 1.158 | 0.839; 1.598 | 0.373 |
| Ischemic stroke |  |  |  |  |  |  |
| Years of follow-up | 0.885 | 0.613; 1.277 | 0.514 | 0.841 | 0.537; 1.317 | 0.449 |
| Age | 1.045 | 0.946; 1.154 | 0.383 | 1.139 | 0.932; 1.392 | 0.203 |
| History of ischemic stroke | 1.973 | 0.192; 20.268 | 0.567 | 2.699 | 0.126; 57.899 | 0.526 |
| History of bleeding | 1.835 | 0.530; 6.348 | 0.338 | 1.215 | 0.190; 7.763 | 0.837 |
| Sample size | 1.000 | 0.9995; 1.0009 | 0.546 | 1.000 | 0.999; 1.001 | 0.953 |
| CHA^2^DS^2^-VASc | 0.946 | 0.404; 2.214 | 0.898 | 0.851 | 0.288; 2.511 | 0.770 |
| Publication year | 1.079 | 0.897; 1.299 | 0.419 | 1.152 | 0.885; 1.499 | 0.294 |
| Major bleeding |  |  |  |  |  |  |
| Years of follow-up | 1.308 | 0.813; 2.105 | 0.268 | 0.979 | 0.693; 1.384 | 0.905 |
| Age | 1.210 | 1.071; 1.369 | 0.002* | 1.202 | 1.036; 1.394 | 0.015* |
| History of ischemic stroke | 0.553 | 0.035; 8.842 | 0.675 | 2.971 | 0.210; 41.944 | 0.420 |
| History of bleeding | 4.136 | 0.989; 17.296 | 0.052 | 2.078 | 0.422; 10.225 | 0.368 |
| Sample size | 1.000 | 0.999; 1.001 | 0.599 | 1.000 | 0.999; 1.001 | 0.465 |
| CHA^2^DS^2^-VASc | 2.655 | 1.034; 6.814 | 0.042* | 1.337 | 0.532; 3.360 | 0.537 |
| Publication year | 1.367 | 1.064; 1.756 | 0.015* | 1.536 | 1.197; 1.971 | <0.001* |
| All-cause mortality |  |  |  |  |  |  |
| Years of follow-up | 1.392 | 0.960; 2.020 | 0.081 | 1.253 | 0.963; 1.630 | 0.093 |
| Age | 1.136 | 1.037; 1.246 | 0.006* | 1.006 | 0.888; 1.140 | 0.920 |
| History of ischemic stroke | 0.652 | 0.067; 6.353 | 0.713 | 2.953 | 0.423; 20.631 | 0.275 |
| History of bleeding | 1.507 | 0.506; 4.489 | 0.462 | 2.034 | 0.675; 6.124 | 0.207 |
| Sample size | 1.001 | 1.0001; 1.0016 | 0.025* | 1.001 | 1.0002; 1.002 | 0.008* |
| CHA^2^DS^2^-VASc | 1.902 | 0.922; 3.921 | 0.082 | 1.300 | 0.702; 2.408 | 0.404 |
| Publication year | 1.263 | 1.065; 1.498 | 0.007* | 1.197 | 1.018; 1.409 | 0.030* |

* p-value <0.05,
Abbreviation: incidence rate ratio (IRR)
